# Yellow card or red card: airport tribulations and new developments on yellow fever vaccination

**DOI:** 10.11604/pamj.2014.17.58.3907

**Published:** 2014-01-26

**Authors:** Raoul Kamadjeu

**Affiliations:** 1 The Pan African Medical Journal, Nairobi, Kenya

**Keywords:** Yellow Fever, yellow card, IHR, vaccination, travel health

## Editorial

As a frequent traveler, the international certificate of vaccination, commonly referred to as the yellow card, has always been the second most important item in my pre-departure check-list, right after the passport. The yellow card is endorsed by the World Health Organization (WHO), issued and stamped by countries health authorities and may be required for entry to certain countries, particularly to travelers coming from countries listed as high risk of yellow fever (YF) by WHO [1]. On arrival, airport health officers with a wide range of expressions on their faces (depending on the mood of the day) will scan the precious document, searching for a stamp and date (proof of where and when the YF vaccination was administered). Sometimes a detailed scrutiny of the card may not be necessary, a peek at the yellow piece before it is even completely unveiled can get you through that check-point.

Why is the yellow card yellow by the way? This translates an obsession with jaundice; the document was originally called the “Certificate of Vaccination or Revaccination against Yellow Fever” and was first issue exclusively for YF as an annex to the International Health Regulation (IHR) (1969). In 2007, a new model (International Certificate of Vaccination or Prophylaxis) came into force with the advent of the International Health Regulation (2005) [2]. The new yellow card was designed to accommodate the increasingly long list of diseases for which proof of vaccination may be required. For most travelers and airport health authorities however, only the YF stamp and date matter.

According to WHO, more than 200,000 cases of YF are still reported every year with 15% of them progressing to a severe form of the illness. YF has no cure and the mortality in the severe forms of the disease can reach 50%. YF vaccination is considered the most effective preventive measure against the disease. Forty-four countries, mostly in sub Saharan Africa and tropical Latin America are considered endemic or at risk for YF virus transmission [3].

Not long ago, the yellow card made news headlines as the source of a near-diplomatic-spat between Nigeria and South Africa; 125 Nigerians where inelegantly expelled from South Africa over an alleged forgery of the document [4, 5]. I have had my own share of scares over the yellow card; forgotten, misplaced, expired. Some of my fellow travelers had it worst; I recently bumped into a colleague at an airport transit terminal; he was on his way back home after being refused access to a country where he was invited for a conference on the reason of expired YF vaccination. The good news is that similar travelers’ miseries may soon be something of the past.

On May 2013, a review of the WHO's Strategic Advisory Group of Experts on Immunization (SAGE) concluding that a single dose of YF vaccination was sufficient to confer life-long immunity against YF disease [6, 7] was made public. This position replaced the previous WHO position on the same (2003) and summarized the latest developments in studies of YF vaccine immunity over time. In light of the SAGE declaration, WHO issued a notice to its member states and associated members; updating the relevant provision of the International Health regulation (IHR) (2005) which previously stated that YF vaccination was only valid for ten years. According to a WHO statement, a resolution on the matter will be submitted in 2014 to the 134th session of the Executive Board and the Sixty-seventh World Health Assembly. This is surely trigger an important policy change in travel health, the most important since the yellow card was introduced more than 40 years ago.

Before you pack for your next travel, remember there is still some grounds to cover before these developments are translated into changes at immigration posts; do not carry an expired yellow card or you may be issued a red one on arrival. To the wise, hello!
